# Unlocking atom-specific radiotherapy – DNA backbone breakage caused by X-ray photoactivation

**DOI:** 10.1039/d5sc03414k

**Published:** 2025-09-11

**Authors:** Pamela H. W. Svensson, Brian Rydgren, Lucas Schwob, Marta Berholts, Bo Stenerlöw, Ouassim Hocine Hafiani, Tomas André, Oscar Grånäs, Nicusor Timneanu, Juliette Leroux, Aarathi Nair, Laura Pille, Bart Oostenrijk, Sadia Bari, Olle Björneholm, Carl Caleman

**Affiliations:** a Department of Physics and Astronomy, Uppsala University Box 516 751 05 Uppsala Sweden pamela.svensson@physics.uu.se carl.caleman@physics.uu.se; b Deutsches Elektronen-Synchrotron DESY DE-22603 Hamburg Germany; c Institute of Physics, University of Tartu EE50411 Tartu Estonia; d Cancer Precision Medicine, Department of Immunology, Uppsala University, Genetics and Pathology Box 256 751 05 Uppsala Sweden; e The Hamburg Centre for Ultrafast Imaging 22761 Hamburg Germany; f Zernike Institute for Advanced Materials, University of Groningen 9747 AG Groningen The Netherlands; g Center for Free-Electron Laser Science, DESY DE-22607 Hamburg Germany

## Abstract

The effectiveness of radiation therapy can be enhanced by understanding the fragmentation mechanisms of iodine-doped DNA oligonucleotide under tender X-rays, as explored experimentally and computationally in our study. By primarily targeting iodine atoms above their L-edge ionization energies, we observed a significant increase in the production of fragments critical to DNA backbone breakage, particularly within mass ranges associated with phosphate and sugar groups. The mass spectroscopy experiments demonstrated that iodine-doped DNA oligonucleotides undergo intense fragmentation at long distances from the initial photoactivation site. Born–Oppenheimer based molecular dynamics simulations confirmed the generation of numerous small fragments, including reactive oxygen species, which are pivotal in enhancing the radiation damage. These findings highlight the effectiveness of iodine doping in amplifying DNA damage in radiotherapy *via* iodine photoactivation, thereby improving the potential for targeted cancer treatment.

## Abstract 2

The effectiveness of radiation therapy can be enhanced by understanding the fragmentation mechanisms of iodine-doped DNA oligonucleotide under tender X-rays, as explored experimentally and computationally in our study.

## Graphical abstract

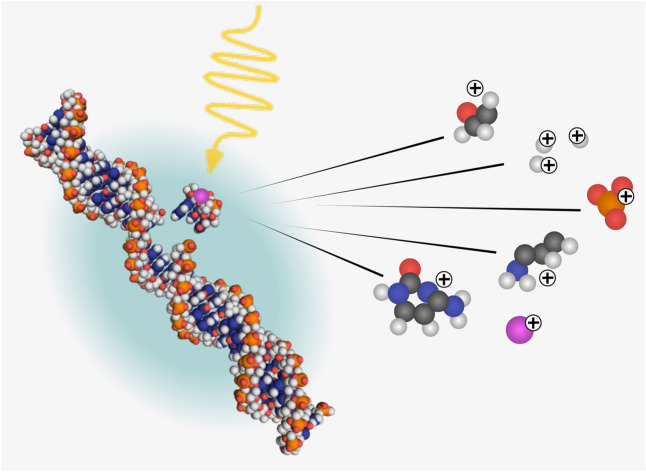

## 1 Introduction

As a leading cause of death worldwide, cancer took nearly 10 million lives in 2020, accounting for approximately one in every six deaths.^1^

**Fig. 1:**
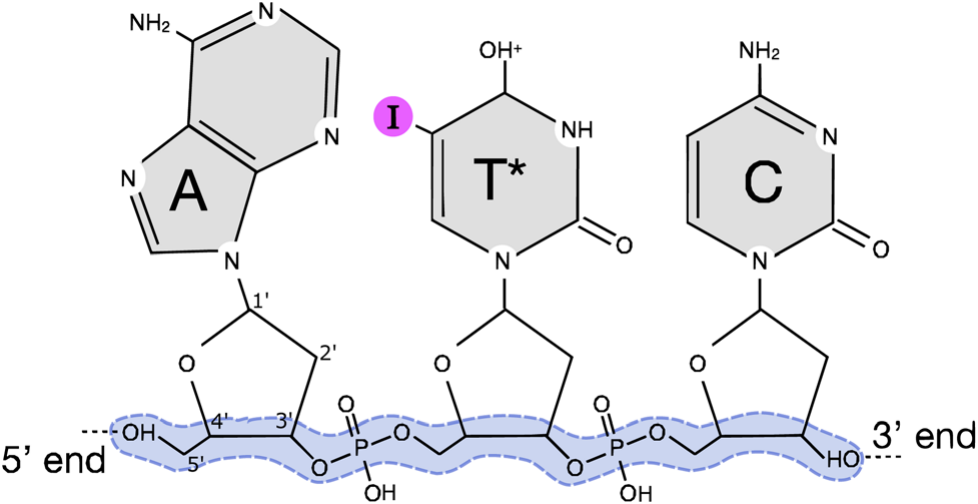
Schematic of the protonated 5′-A/I-dU/C-3′ (AT*C) oligonucleotide. The nucleobases are labelled after adenine (A), thymine (T) and cytosine (C) with a protonation on thymine. An ^127^I atom is incorporated on the thymine base, the halogenated base is therefore noted with an asterisk in the label (T*). Each base is bound to a deoxyribose sugar with the number for each carbon in the sugar labelled. We define the backbone as the chain of covalent bonds shown with a blue highlight. Backbone breakage is thus a scission of at least one of these bonds.

Many cancers can be cured or mitigated if treated effectively, and novel technologies that could enhance cancer treatment are therefore of great interest. Conventional cancer treatment plans often include surgery, chemotherapy, and radiotherapy (RT), or a combination of the three techniques. With RT being given to as many as half of the patients diagnosed with cancer, its efficiency in providing maximum damage to the tumour while minimizing harmful side effects on the surrounding healthy tissue is of great importance.^2^ A promising approach involves increasing the sensitivity of tumour cells to radiation-based therapies by introducing high-Z atoms, thereby accessing a higher photon-ionization cross-section (see Fig. 3) and consequently achieving a more localized energy deposition in the irradiated area. Commonly known as photoactivation therapy. In this study, we explore how the incorporation of a non-radioactive iodine-127 atom (^127^I) into an oligonucleotide affects the fragmentation. We focus on the backbone breakage following L-edge ionization using a combined experimental and theoretical approach. A short DNA oligonucleotide model system was chosen such that the breaking of the backbone could be investigated. The oligonucleotide employed in this study consists of three bases, adenine (A), thymine (T*), and cytosine (C) (5′-A/I-dU/C-3′), with the methyl group in thymine substituted by an ^127^I atom, see Fig. 1. The oligonucleotide will hereafter be denoted as AT*C. The backbone is defined as the chain of covalent bonds that connects the deoxyribose sugar groups with the phosphate groups and is shown in the figure with a blue highlight. The backbone is considered broken if at least one of these bonds is broken.

Heavy elements such as iodine have long been known as candidates for increased radiosensitization in numerous *in vitro* and *in vivo* studies.^3–6^ Iodine can be incorporated into the cancerous deoxyribonucleic acid (DNA) by replacing thymidine with halopyrimidine iododeoxyuridine (IUdR) during the DNA replication process.^7–11^

Some studies have used resonant excitation to higher electronic states or γ-irradiation of the sensitizer agent to induce dissociation when it is incorporated into DNA; however, its efficiency under selective ionization and the following fragmentation mechanisms remain poorly understood.^9,12–15^

**Fig. 2:**
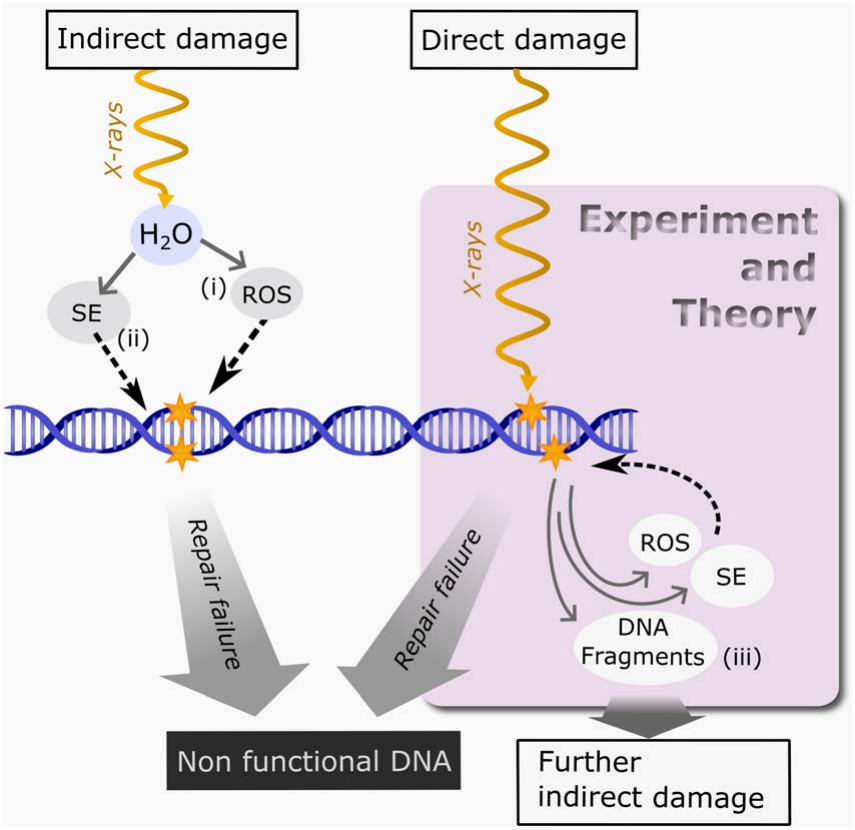
DNA damage from X-ray irradiation, including direct, indirect, and subsequent effects, involves the production of (i) Reactive Oxygen Species (ROS), (ii) Secondary Electrons (SE), and (iii) DNA fragments, which may further disturb cell mechanisms. These agents can further interact with DNA, and if backbone scission is severe, repair mechanisms may fail, threatening cell survival. We conducted experiments and simulations on an oligonucleotide fragmentation caused by direct photoionization damage.

The radiation in RT is commonly delivered using high energy photons which can in various ways induce double-strand breaks (DSB) in the sugar-phosphate backbones of the cancer cell's DNA double-helix as shown in Fig. 2. If the DNA is damaged, directly or indirectly, beyond proper repair, the cells may stop dividing and eventually die. Moreover, DSBs are considered the most difficult type of DNA damage to repair,^16,17^ which is why this study focuses on strand breakage.

A major source of indirect damage is caused by reactive oxygen species (ROS), as shown in (i) in Fig. 2, which are produced through radiolysis of water or energy deposition in biomolecules surrounding the DNA.^18,19^ Of these, the hydroxyl radicals and additional secondary electrons (SE) are the most lethal.^20^ Secondary electrons, as shown in (ii) in Fig. 2, include low energy electrons, that can directly attack DNA components and increase ROS production. Furthermore, the direct damage to DNA can provide another source of ROS and SE, while also producing reactive DNA fragments, (iii) in Fig. 2, which can cause further indirect damage. This study focuses on three aspects in the highlighted box in Fig. 2 as observed in the AT*C oligonucleotide: the direct DNA damage, fragment yield as an indication of DNA backbone breakage, and the generation of reactive species and energetic electrons important for further indirect damage.

Both direct and indirect damage originate in the photon–atom interaction during X-ray exposure of the cancerous tissue. The ionization of a deep shell of an iodine atom incorporated in DNA, followed by Auger–Meitner emission, generates multiple positive charges in the DNA. These mechanisms manifest in a shower of reactive species (including ROS, SE and reactive DNA fragments) whose intensity is scaled based on the amount and energy of ejected Auger–Meitner electrons.^21^ We have in earlier studies managed to benchmark the fragmentation pathways on a molecular level for heavy element doped radiosensitizers (RS) with a two fold effect.^22,23^ In addition to the accumulated high charge state by photoabsorption in the high Z atom, the RSs produce reactive fragment species that contribute to DNA damage. This type of site-selective photoactivation therapy uses the heavy element as a RS, which is activated using X-rays with photon energy tuned to the K-edge of the heavy element.^6,13,24^

It is known that DNA incorporation of radioactive ^125^I isotope atoms *via*^125^I-labelled IUdR produces a high number of DNA strand breaks, where the majority of the non-repairable damage is due to high linear energy transfer of the emitted Auger–Meitner electrons.^25–28^ Thanks to its high efficiency, ^125^I therapy is clinically used to treat *e.g.* prostate cancer.^29,30^ However, using radioactive material inside the body has the disadvantage of delocalised radiation damage in the patient and their close environment.^31^ One solution is to use photoactivation therapy with DNA-incorporated non-radioactive high-Z elements to locally target cancer cells using hotspots provided by the heavy element antennas.^32^

## 2 Method

**Fig. 3:**
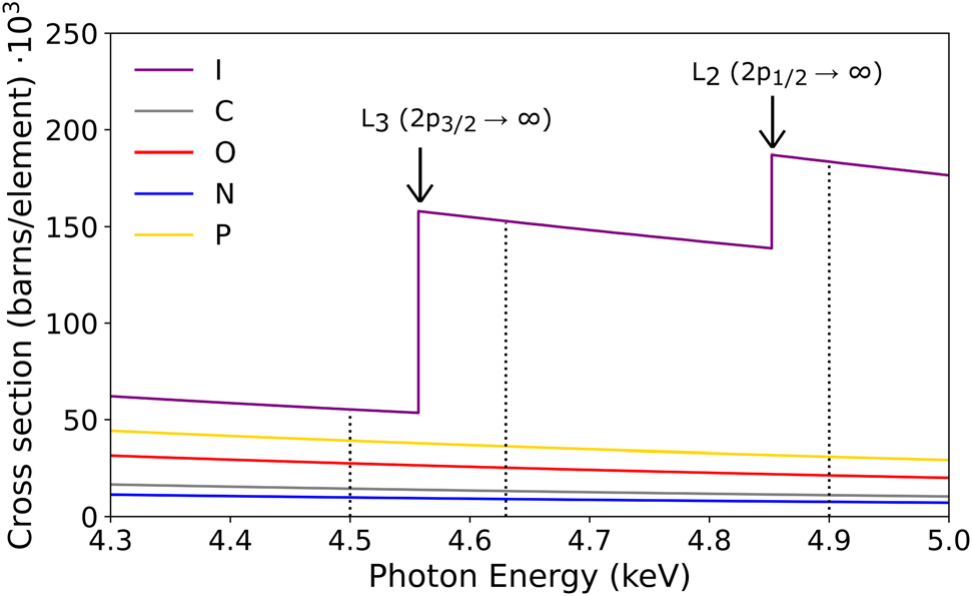
Total cross sections for photoelectronic absorption per element in AT*C with the energies used in the experiment indicated as dotted lines (4500, 4630 and 4900 eV). The cross sections per element have been multiplied by the number of atoms of that element in the measured oligomer (1 I, 28 C, 16 O, 10 N, 2 P) such that the total cross sections per element in the molecule can be compared. At photon energies of 4630 and 4900 eV, the photoabsorption cross-section is mainly dominated by the iodine atom.

To study the photoactivated molecular fragmentation of DNA doped with iodine, a short single-stranded oligonucleotide was chosen as a model system. The iodine was attached to the thymine in the oligonucleotide base due to the established efficiency of IUdR as a chemical modifier and the prevalence of thymine, which constitutes approximately one-third of the human genome. By doping this base we can potentially integrate a higher number of RSs into the DNA *via* IUdR in contrast to halogenated cytosine or guanine analogues.^33^ This approach allows us to maintain a high yield of Auger–Meitner electrons and increased damage localization, while avoiding the use of unstable isotopes. K-edge energy of iodine is situated around 33.2 keV which can be produced using a compact synchrotron X-ray source, making treatment at such photon energies accessible in future hospital setups.^34,35^ The primary de-excitation pathways from core-holes in the I K-shell are usually through radiative K_α_ to the L-shell which in turn produces an Auger–Meitner cascade. This relaxation happens within a few femtoseconds, a time period which is much faster than nucleic motion. Therefore, a photoinduced fragmentation using iodine L-edge ionization energies is a comparable method to study fragmentation pathways and generated fragments at K-edge energies. By primarily targeting the iodine atom close to its L-edge resonance energies, 4630 and 4900 eV, the photoionization cross-section is dominated by the iodine atom. Fig. 3 shows more than 75% of the photons are absorbed by the iodine atom. The following fragmentation is thus mainly caused by photoabsorption and subsequent decay cascades at the iodine site while absorption on the other, lighter, atoms remains low. Moreover, ionization of I 2p electrons results in ≈4 times higher charge states than ionization of core-levels in the lighter elements which make up most of the tissue.^36^

### 2.1 Experiment

The experiment was done by interfacing a home-built tandem mass spectrometer to the P01 beamline of the PETRA III synchrotron facility at the Deutsches Elektronen-Synchrotron DESY.^37^ The oligonucleotide AT*C was purchased from Integrated DNA Technologies, BV and used without further purification. The sample was diluted in 50 : 50/water : methanol solution to 25 μM, and 1% of formic acid was added to favour protonation of the oligonucleotide. Briefly, the sample was injected using an electrospray ionization source (ESI) and the positively charged ions were thereafter transferred through a capillary and further focused and guided with a RF funnel and octupole ion guide. The singly protonated parent molecules at charge +1 were filtered by their mass/charge (*m*/*q*) using a mass filter and subsequently accumulated into an ion trap.

A helium buffer gas was used to decrease the kinetic energy of the ions down to room temperature. The molecules were exposed to the photons from the beamline and ionized in the ion trap and the produced fragments were measured with a time-of-flight mass spectrometer.^38^ To study the effect of iodine ionization of the L-shell, mass spectra of the parent ions and their ionic fragments were collected at three photon energies around the I L-edge. Below the ionization edge (4500 eV), above the I L_3_-edge (4630 eV) and I L_2_-edge (4900 eV). Three mass spectra per photon energy were collected in three steps to account for all types of background ions at a mass range of *m*/*q* 60 to singly protonated parent mass *m*/*q* 957.5. In the first step, with the photon beam off, the precursor ion intensity of the ESI is measured in a mass spectrum with small contributions of fragments originating from collisions with the buffer gas, [MS_no photons_]. In the second step, the photon beam is turned on and a mass spectrum from photo-induced fragmentations in the ion trap is recorded, [MS_photons_]. In the third step in the cycle, the ESI source is turned off while the photon beam remains on and a background mass spectrum is collected from photoabsorption by the residual gas, without any sample in the interaction region, [BG]. The mass spectra presented in Fig. 5a are thus time-of-flight data, converted to *m*/*q* resulting from subtraction involving these three mass spectra [MS_no photons_], [MS_photons_] and [BG] according to:

1 Subtracted data = [MS_photons_] − [MS_no photons_] − [BG]

The spectra were normalized to the area of the parent peak recorded in the [MS_no photons_]. To account for fluctuations in the ESI source, a hundred cycles of data accumulation were performed with synchrotron light exposure of 10 seconds per mass spectrum.

### 2.2 Theory

The features of detected fragment peaks in the experimental mass spectra are the result of individual molecular dissociations occurring during several microseconds before the atomic systems can reach the detector. Drawing conclusions from dynamics occurring at femtoseconds time scale after light exposure using data collected several microseconds later is cumbersome, and potentially impossible due to the information loss at such long detection time scales. We have therefore turned to Born–Oppenheimer based molecular dynamics to model the protonated DNA oligonucleotide at a charge state obtained after photoionization in the experiment. We have used the Siesta programme with exchange and correlation treated by the Generalized Gradient Approximation functional within the Perdew–Burke–Ernzerhof (PBE) parameterization.^39^ We employed a scheme of dynamics simulations which have proven successful in replicating the fast dissociation mechanisms due to core-level ionized molecular systems.^40^ Hydrogens were added to the phosphate groups to account for possible protonation sites and sufficient agreement with the mass of the molecule as recorded in the experiment.

To create starting structures for our simulations that represent the entire configuration space would be time consuming using our Born–Oppenheimer-based molecular dynamics approach. In earlier studies (with smaller molecules than we simulate here) we have done pre-simulations to sample confirmation state of around 1 ps, and these simulations have shown good agreement to experiments.^22,23,41^ In the present study we have expanded the pre-simulations to 10.5 ps by doing the following. Starting from one simulation in ground state (+1) we simulated 0.5 ps, and from that first trajectory we pick five structures, 0.1 ps apart. These structures were then used as new starting structures, and new velocities based on the temperature were assigned to the atoms, and simulated for a total of 2 ps. This emulates the molecular dynamics occurring in the experiment chamber before synchrotron light irradiation. From these five independent trajectories, 100 snapshots were selected with at least 100 fs apart to ensure a variety of geometrical conformations which are used as input in the next step. Since the ionization event and subsequent emittance of photo and Auger–Meitner electrons occur at a much shorter time scale than nuclear motion, we have concluded in previous studies that the fragmentation dynamics is mainly mandated by the electronic structure given by the ground state geometry. The total charge is set to +9 or +3 depending on I 2p ionization or C, N, O, S 1s ionization for a singly protonated system and with the additional charges coming from Auger–Meitner decay. Except the most probable charge state +9, iodine 1s ionization can lead to other final charge states as have been shown in the literature.^32,36,42^ 100 simulations for charge states +9 and +3 were performed for the iodinated oligonucleotide, and an additional 100 simulations for the non-iodinated system at +3 charge. To verify the accuracy in choosing charge state +9, we performed supportive simulations at charge +8 and +10, see Fig. S3. They indicate that the dominating fragmentation pathways are not affected by a large extent if the charge on the system is ±1 which would correspond to a change in charge per atom by ±0.01*e*. The resulting fragmentation dynamics from the simulations were observed for 1 ps with a time step of 0.5 fs. To account for electronic energy increase in the oligonucleotide due to Auger–Meitner decay, and to observe the bond instabilities which can only be observable at longer time scales, the temperature was set to 5000 K, or ≈40 eV. This ensures a sufficient amount of time for fragment formation while lowering the computational cost. To quantify the integrity of the covalent bonds in the atomic system, we employ Souvatzis bond integrity parameter;^40^
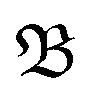
2
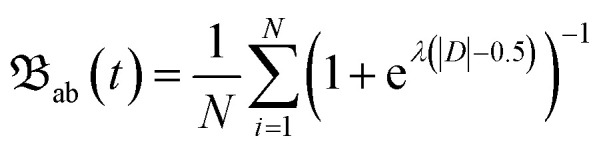
where*D* = *d*_*i*ab_(*t*) − *μ*(*d*_ab_^*T*^(*t*)) − *σ*(*d*_ab_^*T*^(*t*))with distance *d*_*i*ab_ between atoms a and b in the production simulation, distance *d*_ab_^*T*^ between atoms a and b in the presimulation, and a smearing parameter *λ*.^41^ Fragments were allowed to form new bonds during the simulation if the distance between two atoms was lower than 1.8 Å. By assuming negligible effects on the molecular geometry during electronic relaxation, we start each simulation with the multiple charged oligonucleotide in its electronic ground state. The generated fragments are recorded at the final time step using the parameter. The charge of the observed fragments in the simulations are unknown and their mass will therefore be referred to by their unified atomic mass units (u).

## 3 Results and discussions

It is medically shown that it is possible to introduce iodine into the DNA by the introduction of the IUdR molecule.^4,8,13,43^ In the following results we explore the fact that the ionization cross section for iodine above the iodine L-edges (4630 and 4900 eV) is higher than for any of the other atoms in the DNA (Fig. 3), and we can therefore primarily ionize iodine in the AT*C molecule. Since the dominating decay pathways after iodine K-edge is *via* X-ray fluorescence and the production of a hole in the L-shells, we can model the radiation damage indirectly *via* ionization of L-shell electrons.

Fig. 5 shows fragments recorded in the experiment within the mass range *m*/*q* 60–140 at three different photon energies. Highlighted regions show fragments originating from breaking the backbone, as defined in Fig. 1, which are validated by theoretical simulations. In all spectra we see generally the same types of fragments being formed. However, despite that the methyl group substituted by ^127^I in the thymine base is relatively far away from the backbone, we observe a general increased intensity in the production of fragments related to breakage of the backbone within the mass range of *m*/*q* 60–140. Notably, fragments critical for backbone stability, such as PO_4_^+^ and PO_3_^+^ ions, have a stronger signal above the iodine L2-edge from bond scission at the deoxyribose 5′ and 3′-carbon. Fragments containing phosphorous are solely coming from the backbone and act as fingerprint for DNA backbone breakage.

Other peaks which increase with the higher photoionization cross section at these photon energies, belong to the sugar group (C_4_H_*x*_O^+^), where *x* = {1, 2, 3…7}, *i.e.* fragments within the range *m*/*q* 65–71. These peaks are clustered together depending on the number of covalently bonded hydrogens. This mass range, also coincides with fragments observed from base damage as has been shown at lower photon energies.^44,45^ The similarities are especially prominent within the same mass range as the sugar group for cytosine and thymine without the methyl group (also known as uracil). To support the experiment we have simulated the generation of small fragments at masses below *m*/*q* 61, and the origin of fragments at certain peaks in the experimental mass spectra using Born–Oppenheimer based molecular dynamics as described in Subsection 2.2. The simulations suggest that the peaks at *m*/*q* 65, 68, 69 and 71 are indeed mainly originating from backbone-specific fragments after deep core level ionization (final charge +9), as shown in Fig. S2 in the SI. The dominant peak in the mass spectra can be noted at *m*/*q* 81, likely resulting from a combination of ions originating from different sources. We assign *m*/*q* 81 to the PO_3_H_2_^+^ fragment since our simulations in Fig. S2 show only a weak contribution from the C_5_H_5_O^+^ fragment. For symmetry reasons, the fragment C_5_H_5_O^+^ should show neighbouring peaks with a similar relative clustering intensity as the sugar group between *m*/*q* 65–71.

For certain peaks, such as *m*/*q* 69, 81, 108, and 112, the peak area decreases above L_3_-edge, and then again increases above L_2_-edge. One could expect that the total number of measured fragments should increase with higher cross-section. However, ionization of I 2p also increases the production of highly charged species, the probability increases therefore for large fragments to dissociate to smaller fragments, both within and outside the detectable mass range of the experiment. Although the peak intensities vary, overall the same types of fragments are produced at each photon energy. Furthermore, we see an increased release of singly charged iodine ions at *m*/*q* 127 from ionization at higher photon energies. The peak intensities, matching the photon-iodine absorption cross-section shown in Fig. 3, support the site selectivity of predominantly ionizing iodine at energies above the iodine L3 and L2-edge. The base damage recorded as fragments at *m*/*q* 66, 67 and 70 are thus potentially originating from T* base due to the close vicinity of the photoabsorption site. A release of A, C and non-iodinated T* bases are detected in both their protonated and non-protonated form similar to the non-iodinated oligonucleotide measured in an earlier experiment.^46^ These peaks are not highlighted since these fragments do not guarantee the rupture of the backbone. Heavier ions, aside from nucleosides and free iodine atoms, are nearly absent in the experimental spectra.

**Fig. 4:**
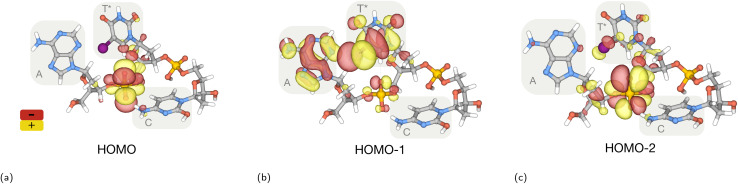
Orbitals belonging to the valence states of the AT*C oligonucleotide in its ground state. (a) HOMO is delocalised around the phosphate group between bases A and T* with main contributions from the O p-orbitals. (b) The HOMO−1 is mainly delocalised across the T* and A bases, with only a small contribution from the backbone. (c) Is strongly localised on the phosphate group between nucleobases A and T*.

These results indicate the efficient cleavage of bonds outside the vicinity of the iodine photoabsorption site. This widespread effect is primarily attributed to the detection of free neighbouring bases from the scission of the N–C1′ bond between the base and the corresponding deoxyribose sugar. This suggests a rapid charge migration from the iodine atom in the thymine base to the backbone. We can from our simulations at charge state +9 identify five sites in the backbone where the scission are the most prominent. In Fig. 6a the percentage of a bond breakage is shown with a blue scissor. Both phosphate groups have a weaker P–O bond in the 3′ direction of the backbone with a breakage in 82 and 70% of the simulations. Following the scission trends seen in the fragmentation pathways, we observe that this weakening is an indicator of core holes in the valence orbitals far from the original ionization site. The P-containing fragments produced from scission in the 5′ direction from the T* base are formed at short timescales, ≈20 fs after ionization. This indicates bond break from reordering of the electron cloud. In the 3′ direction from the T* base, P-containing fragments are formed at much longer time scales. The deoxyribose sugar bonded to base C has a scission of the C3′–C4′ bond in 80% of the simulations resulting in opening of the ring. This process, in combination with the remaining structures from the release of P-containing fragments, enables the formation of intermediate carbon-based fragments like C_2_H_4_O. These fragments are unstable and often dissociate and produce other reactive fragments, such as the reactive hydroxyl ion. We can furthermore observe that the stability of the deoxyribose sugar, bonded to the adenine base, stimulates the release of non fragmented adenine base less than 30 fs after ionization. In a second step, the deoxyribose sugar is released. An example of this type of fragmentation pathway is shown in Fig. S2. To gain a deeper understanding about the mechanisms behind, we have calculated the three highest occupied molecular orbitals (HOMO) and included visualizations of them in Fig. 4. They show antibonding character mainly in the phosphate groups, but more bonding character in the bases. This could explain the release of intact bases while effective scission into the sugar-backbone.

**Fig. 5:**
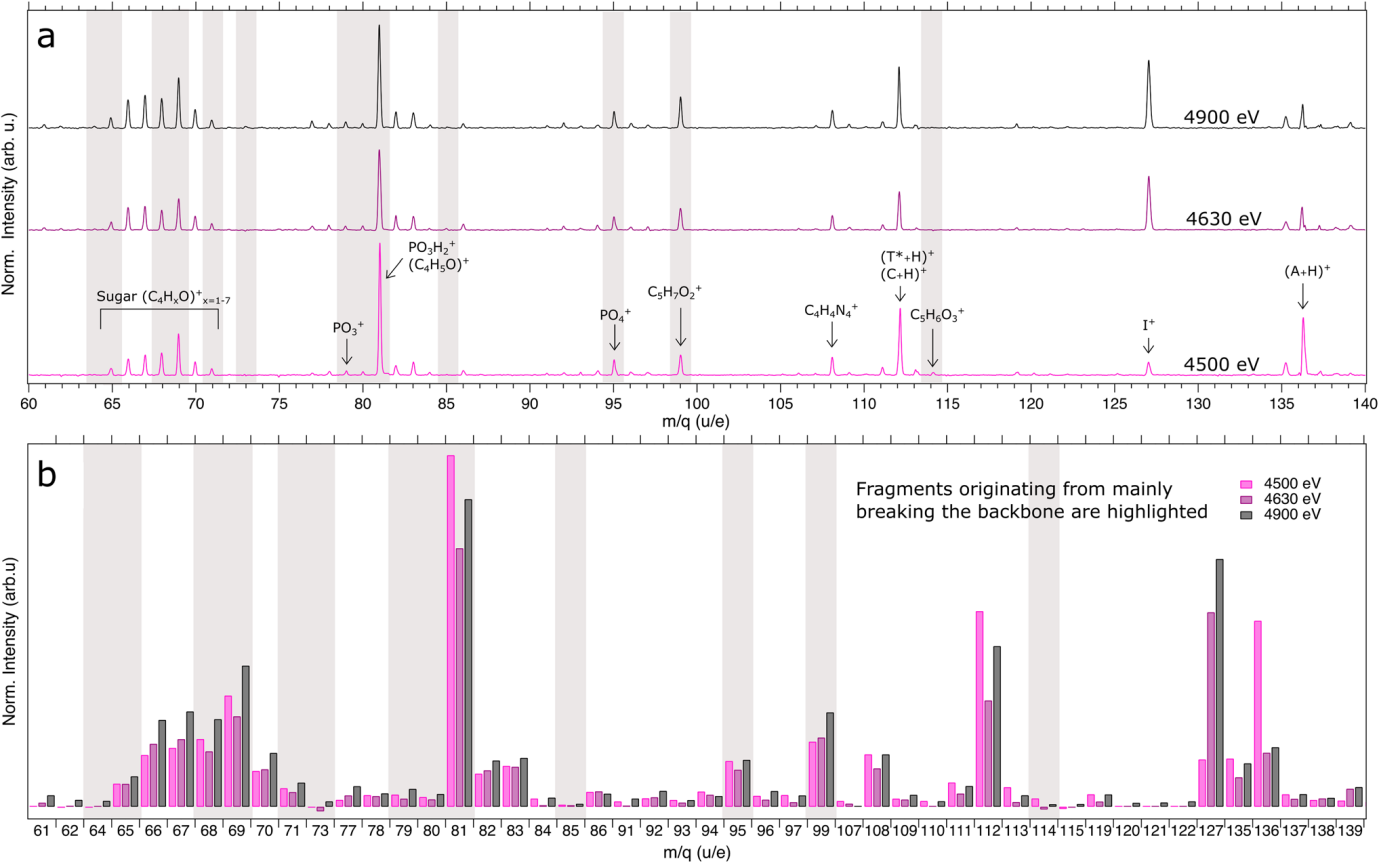
(a) Measured mass spectrum for AT*C for three incident photon energies at 4500, 4630 and 4900 eV within the mass range of *m*/*q* 60–150. Peaks that contribute to the breakage of the sugar-phosphate backbone are emphasized in gray. (b) Integrated areas of the main peaks from the spectra above.

**Fig. 6:**
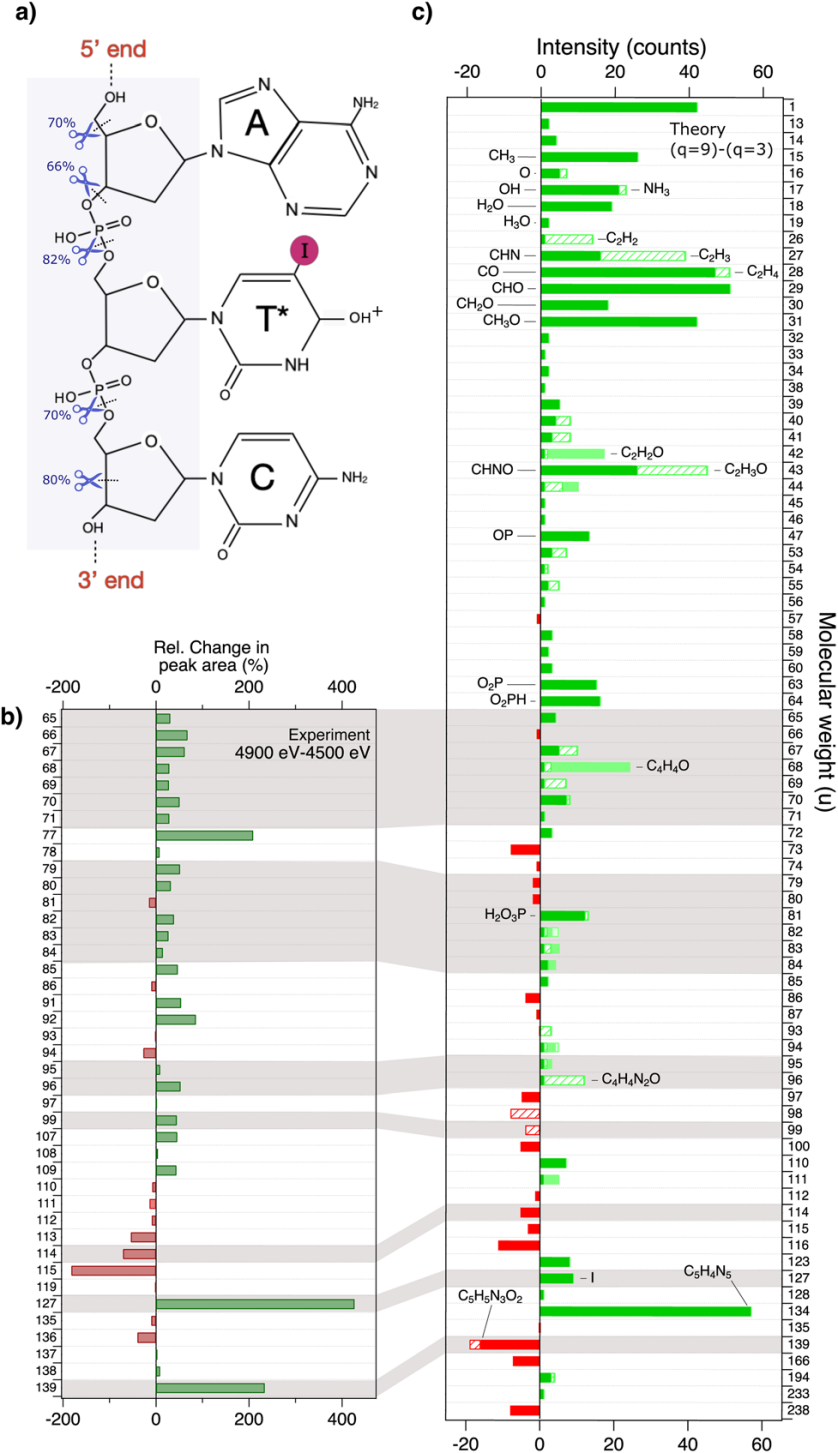
(a) Structure of the protonated, iodinated oligonucleotide AT*C. The most prominent bond breaks recorded in simulations of the backbone at charge state *q* = +9 are shown. (b) Relative change in peak areas between 4500 eV and 4900 eV of the strongest peaks observed in the experimental mass spectra in Fig. 5. An increase in peak area is shown in green and a decrease is shown in red. (c) The change in the number of fragment occurrences from simulations above (*q* = +9) and below (*q* = +3) I 2p ionization. The molecular weight can represent different fragment species and the bar is therefore divided with different fills for different species. Some theoretical features in Fig. 5c) can be compared to the experimental data in Fig. 5b) as shown highlighted in gray. Emphasis is laid on the mass range below *m*/*q* 65 in the theoretical bar plot showing a high production of small fragments.

In the experimental spectra we see several peaks that are caused by fragments containing P, most dominating is the PO_3_H_2_^+^ peak. These P-containing fragments can only be created if the backbone is broken, which is desirable. From Fig. 6a we see that the P link between the A and the T is more likely to break than the P link between C and T. The mechanism behind this is not trivial to understand, but can be illustrated by the visualization of the three highest molecular orbitals (HOMO, HOMO−1 and HOMO−2). Assuming that initial ionization occurs on iodine 2p, the vacancy will be filled within femtoseconds through Auger–Meitner decay, resulting in a molecule with vacancies in the valence band. By calculating the three highest occupied molecular orbitals, we can get a hint to why the P link between A and T is the weakest part of the backbone. Our calculations show that this specific link contributes to all three highest orbitals, which in turn indicates that this bond will be weakened if we start removing valence electrons from the molecules. This weakening also supports why P-fragments originating from the 5′ end appears to form faster than P-fragments from the 3′ end.

The shift from the production of heavy fragments to lower mass fragments when mainly targeting iodine ionization below and above L-edge energies becomes apparent. This is evident when comparing the relative change in the peak area between 4900 eV and 4500 eV as shown in Fig. 6b. Simulations complement these results in Fig. 6c. Here we compare the change in produced fragments between net charge +9 (I 2p ionization) and +3 (1s ionization of the lighter elements, C, N, O, and P in DNA).

The production of fragments related to the sugar group during the experiment increased on average by 43% and we see an increased production of backbone relevant species at *m*/*q* 79, 95 and 99 from photoactivating the iodine atom. Furthermore, amongst the heavier fragments, it is only single iodine ions at *m*/*q* 127 and IC^+^ ions at *m*/*q* 139 which increase dramatically in production by 430% and 230% respectively. This effect is to be expected due to the high charge state of iodine atom before charge migration to other parts of the oligonucleotide.

The main trends in the theoretical results presented in Fig. 6c follow the experimental data, such as the increase in production at masses *m*/*q* 65–71, 95–96 and 127 above I L-edge. By using a theoretical approach of simulating the fragmentation dynamics of the oligonucleotide beyond experimental limits, it is clear that a vast amount of dissociative ions are expected to form at molecular weights below the experimental detection limit of *m*/*q* 65. Several fragments belonging to the phosphate-group from breaking the backbone are recorded at masses 47, 63 and 64 u. At masses between 16–19 u we can distinguish relatively high counts of ROS and amongst them, the hydroxyl group, which is of interest to increase lethal effect in a solvated DNA system. In the range 26–31 u we have high counts of carbohydrate derivatives mainly originating from sugar-backbone breakage. The results from the simulations confirm the hypothesis of charge transfer from the photoabsorption site of the iodine, to the rest of the molecule. Additionally, the covalent bonds in the sugar-phosphate backbone break before the bonding instabilities from the high charge state can affect the adjacent bases. Therefore, the bases A, C and non-iodinated T* are still produced at relatively high counts in both experiment and in simulations. The full record of fragments observed in the calculations are available in SI.

## 4 Conclusion

From a combined experimental/theoretical approach, we have studied the fragmentation of a protonated DNA oligonucleotide upon photoactivation of an incorporated iodine atom at applicable energies which can be used in radiotherapy. The resulting dissociation of the oligonucleotide trimer in the experiment setup leads to severe breakage of the sugar-phosphate backbone in four main locations in conjunction with fragment species from base damage. A majority of the detected fragments are produced with higher yields during X-ray exposure close to the iodine L_2_ resonance specific energy. Moreover, the overall intensity of the fragments increases at this energy, suggesting intense breakage due to high charge states otherwise unattainable without iodine doping. Using simulations, we can also observe high counts of small fragments within a group belonging to the radiotherapy-important family of ROS molecules and fragments originating from the backbone. Due to the high charge state imposed by iodine L-edge ionization, it is likely that many of these fragments are ions and are thus highly reactive in close vicinity to their production site. The mentioned observed fragments in combination with secondary electrons and photons provide a background for understanding why iodine-doping of DNA becomes an efficient cell killer of cancerous tissue.

Water molecules are known to affect charge transfers *via* interatomic coulombic decay (ICD) and electron transfer mediated decay (ETMD) electrons,^47,48^ or solvated electrons.^15^ This can be both from the molecule to the surrounding,^49^ or from the surrounding to the molecule.^50^ These processes play a role in redistributing positive charge following deep inner shell ionization. The ultrafast charge delocalization from the ionization site over the whole molecule is a critical step in determining fragmentation outcome. Moreover, the fragmentation dynamics may be slowed down due to solvent molecules impeding the dissociative nuclear motion and some data suggests increased stability of solvated DNA.^51,52^ Additionally, the presence of water can induce conformational changes, further modulating the potential energy surface and altering dissociation pathways. These pathways are non trivial to access experimentally but Liquid Chromatography-Mass Spectrometry (LC-MS) studies on systems similar to ours, in solution, have shown that incorporating an iodine into the DNA can increase the radiation damage.^15^

However, further studies are thus needed to understand the intricate behaviour of radiation damage of DNA in an environment closer to its native state in a living organism. A solvated system could potentially lead to other ionization pathways within the DNA strands; however, the intense charge accumulation from the Auger–Meitner cascade from the iodine would still generate a highly concentrated charge which can amplify the production of reactive species. Additional studies are also needed to understand how localized the damage is in iodinated oligonucleotides with much longer lengths and with the inclusion of a second strand. In summary, our study shows that the introduction of stable isotope ^127^I cause more backbone breakage and higher production of ROS molecules when tuning the ionizing radiation to energies where the photoabsorption cross-section is dominated by the iodine atom in the iodine-doped DNA. The local radiation damage hotspots from iodine can lead to a more effective radiation treatment than conventional methods currently available. By using selective energies in combination with iodine doping, the RT practitioner can amplify the dosage in the tumor and mitigate unnecessary dosage in surrounding healthy tissues. This approach can reduce side effects and enhance patient outcomes, ultimately saving more lives.

## Author contributions

Experiments, PHWS, LS, MB, TA, JL, AN, LP, BO, SB; computation and software, PHWS, BR, OG, CC; experimental data analysis, PHWS, MB, OHH; calculations analysis, PHWS, BR, OG, NT, CC; writing of the manuscript, PHWS, CC; review and editing, all authors; funding acquisition; SB, OB, CC.

## Conflicts of interest

There are no conflicts to declare.

## Supplementary Material

SC-OLF-D5SC03414K-s001

## Data Availability

The main software, Siesta is available from https://siesta-project.org/. The data supporting this article have been included as part of the SI, or can be requested from the authors. Supplementary information is available. See DOI: https://doi.org/10.1039/d5sc03414k.
