# Supplementary material for: Unlocking atom-specific radiotherapy – DNA backbone breakage caused by X-ray photoactivation
Source: Chem Sci. 2025 Sep 11;16(41):19235–43. doi: 10.1039/d5sc03414k (PMC12445220; doi:10.1039/d5sc03414k)
Supplement: SC-016-D5SC03414K-s001 [file SC-016-D5SC03414K-s001.pdf]

Supplementary Information (SI) for Chemical Science.
This journal is © The Royal Society of Chemistry 2025

Supplementary material: Unlocking atom-specific radiotherapy - DNA backbone breakage caused by X-ray photoactivation

Pamela H.W. Svensson,[1] Brian Rydgren,[1] Lucas Schwob,[2,3] Marta Berholts,[1] Bo Stenerlöw,[4] Ouassim Hocine Hafiani,[1] Tomas André,[1] Oscar Grånäs,[1] Nicusor Timneanu,[1] Juliette Leroux,[2,3] Aarathi Nair,[2,3] Laura Pille,[2,3] Bart Oostenrijk,[2,3] Sadia Bari,[2,3] Olle Björneholm,[1] and Carl Caleman[1,5]

[1] *Department of Physics and Astronomy, Uppsala University, Uppsala, Sweden*
[2] *Deutsches Elektronen-Synchrotron DESY, Notkestr. 85, 22607 Hamburg, Germany*
[3] *Deutsches Elektronen-Synchrotron DESY, DE-22607 Hamburg, Germany*
[4] *Cancer Precision Medicine, Department of Immunology, Genetics and Pathology, Uppsala University, Uppsala, Sweden*
[5] *Center for Free-Electron Laser Science, DESY, DE-22607 Hamburg, Germany*

We have performed experiments and Born-Oppenheimer based molecular dynamics on the halogenated oligonucleotide AT*C. The full mass spectra obtained during the experiment are shown in Fig. S1. The measurement was optimized to ensure that a sufficient number of parent molecules reached the ion trap, while still allowing for the observation of lighter fragments. To obtain maximum radiation damage in a tumour one aims to break both strands in the DNA of the cancerous tissue, therefore we focus on produced fragments which contain at least one of the atoms from the sugar-phosphate chain as defined in the paper. The full record of fragments which fulfill this statement below iodine L-edge (net charge +3) and above iodine L-edge (net charge +9) is shown in Table SI. A fragmentation pathway at charge +9 is shown in Fig. S2.

In addition to the +9 iodinated oligonucleotide simulations corresponding to photon energy 4900 eV in the experiment, we have performed simulations of both the non-iodinated AT*C and iodinated molecule at charge state occurring below I 2p ionization, corresponding to photon energy 4500 eV in the experiment. At this photon energy the cross-section is dominated by the lighter atoms in the oligonucleotide. After ionization, the decay primarily involves the emission of an Auger-Meitner electron, which, together with protonation, results in a final charge state of +3. The full record of observed fragments are shown in Table SII, SIII, SIV and SV. In summary, we see generally similar trends amongst the observed fragments at +3 charge without iodine as with iodine with the main discrepancy being a much higher production of hydrogens and a 25% increase in production of $H_6C_5N_5$ fragment at mass 136 u from ionizing the non-iodinated oligonucleotide. Furthermore, the iodinated oligonucleotide dissociated generally to slightly heavier fragments (mainly due to inclusion of heavy atom iodine). Since the intensity and species of fragments produced from ionizing the non-iodinated and iodinated oligonucleotide at the same charge state are comparable, we validate our assumption that low mass atoms are dominating below the I L-edge. A record of the input-files and parameters used for the simulations are available in *DOI: 10.5281/zenodo.13846820.*

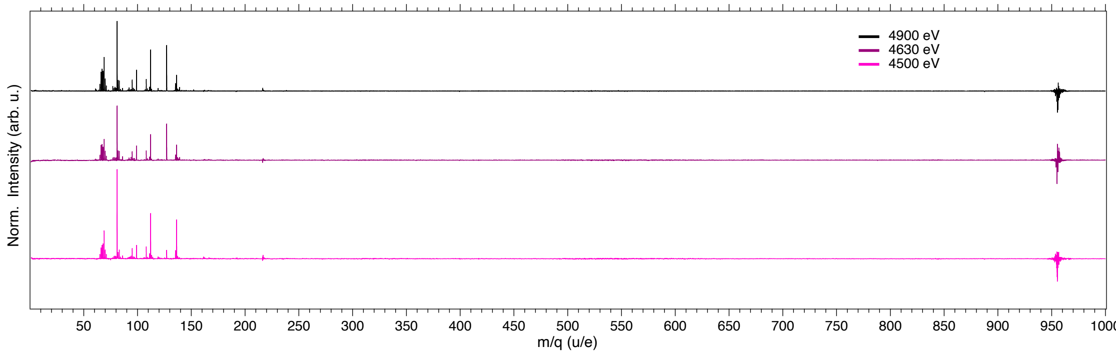

Figure S1: Full spectra of protonated AT*C molecule at energies 4500, 4630 and 4900 eV. The non-fragmented protonated oligonucleotide is located at m/q 957.5. A small feature is noted at m/q 216 but after integration of the peak, the total area was equal $\approx 0\, mI/q$ and is thus excluded from Figure 5. This peak can be assigned to the adenine base + sugar moiety.

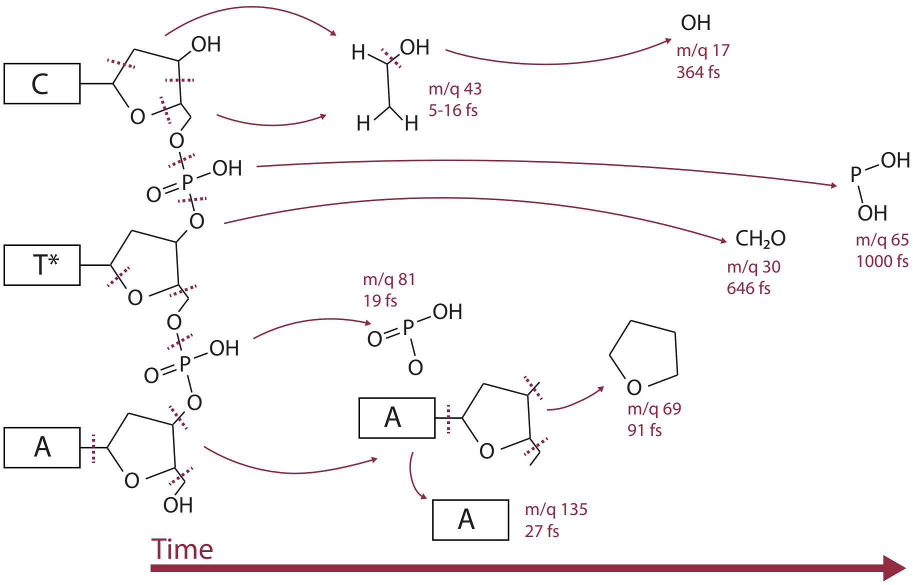

Figure S2: An example of observed fragmentation pathways from simulated fragmentation of the AT*C oligonucleotide at charge +9. Timescales in fs for fragmentation formation is also indicated. ROS species such as the OH fragment were observed as secondary species after the intermediate fragment C_2H_4O at m/q 43 in this example.

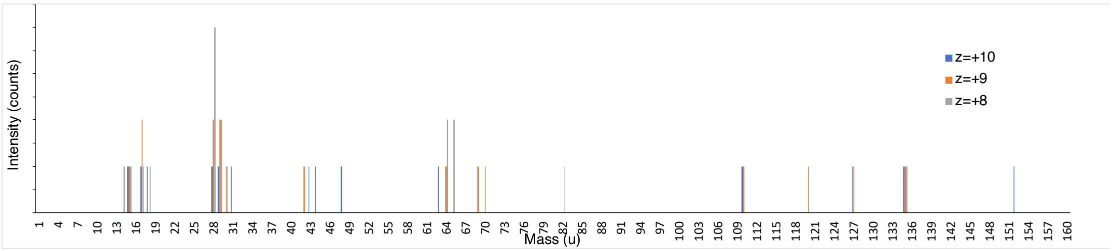

Figure S3: Comparison of mass spectra gained from three independent simulations for the same starting structure at different charge states +8, +9 and +10. Similar clustering is noted around mass 29u, corresponding to fragments like CO and COH. Also a cluster at mass 17u corresponding to CH_3, OH, and H_2O fragments. All three charge states also produce free adenine bases at mass 135u.

Fragment	mass (u)	+3 (NoI)	+9 (wI)
CH_3	15.0	1	8
HO	17.0	2	7
H_2O	18.0	5	16
C_2H_2	26.0	3	10
C_2H_3	27.0	2	15
CO	28.0	22	41
C_2H_4	28.1	1	3
CHO	29.0	12	45
CH_2O	30.0	44	60
CH_3O	31.0	15	57
C_3H_3	39.1	-	5
C_3H_4	40.1	-	4
C_3H_5	41.1	-	5
C_2H_2O	42.0	6	22
C_2H_3O	43.0	10	31
C_2H_4O	44.1	18	24
C_2H_5O	45.1	3	2
OP	47.0	3	16
C_4H_5	53.1	-	4
C_3H_3O	55.1	2	5
C_3H_4O	56.1	4	5
C_3H_5O	57.1	4	3
$C_2H_2O_2$	58.0	6	9
$C_2H_3O_2$	59.0	1	3
$C_2H_4O_2$	60.1	1	4
O_2P	63.0	4	19
HO_2P	64.0	26	42
H_2O_2P	65.0	7	11
C_4H_4O	68.1	10	31
C_4H_5O	69.1	6	12
C_4H_6O	70.1	8	3
C_4H_7O	71.1	6	3
$C_3H_4O_2$	72.1	2	5
$C_3H_5O_2$	73.1	10	2
O_3P	79.0	6	4
HO_3P	80.0	33	31
H_2O_3P	81.0	10	22
H_3O_3P	82.0	3	4
C_5H_7O	83.1	3	1
$C_4H_5O_2$	85.1	2	4
$C_4H_6O_2$	86.1	5	1
$C_4H_7O_2$	87.1	3	2
H_2O_4P	97.0	6	1
H_3O_4P	98.0	2	2
$C_5H_6O_2$	98.1	9	1
$C_5H_7O_2$	99.1	16	12
$C_5H_8O_2$	100.1	7	2
CH_4O_4P	111.0	2	3
$C_5H_6O_3$	114.1	8	3
$C_5H_7O_3$	115.1	4	1
$C_5H_8O_3$	116.1	13	2
$C_2H_4O_4P$	123.0	1	2
$C_2H_4O_5P$	139.0	3	-
$C_7H_8N_3O_2$	166.2	7	-
$C_5H_8O_5P$	179.1	1	3
$C_5H_9O_5P$	180.1	4	1
$C_8H_9N_3O_3$	195.2	3	-
$C_5H_8O_6P$	195.1	3	-
$C_5H_9O_6P$	196.1	3	2
$C_9H_9N_5O$	203.2	4	1
$C_{10}H_{11}N_5O_2$	233.2	6	-
$C_{10}H_{12}N_5O_2$	234.2	3	-
$C_5H_{10}O_9P_2$	276.1	3	-
$C_7H_6N_2O_3I$	293.0	1	4
$C_8H_6N_2O_3I$	305.1	3	1
$C_9H_{10}N_2O_7PI$	416.1	3	-

Table SI: Table of generated fragments originating from the sugar-phosphate backbone observed (at more than 2 occasions) at C K-edge energies (net charge +3) and I L-edge energies (net charge +9) in Born-Oppenheimer based Molecular Dynamics calculations. The numbers represent the number of simulations that a fragment appears in.

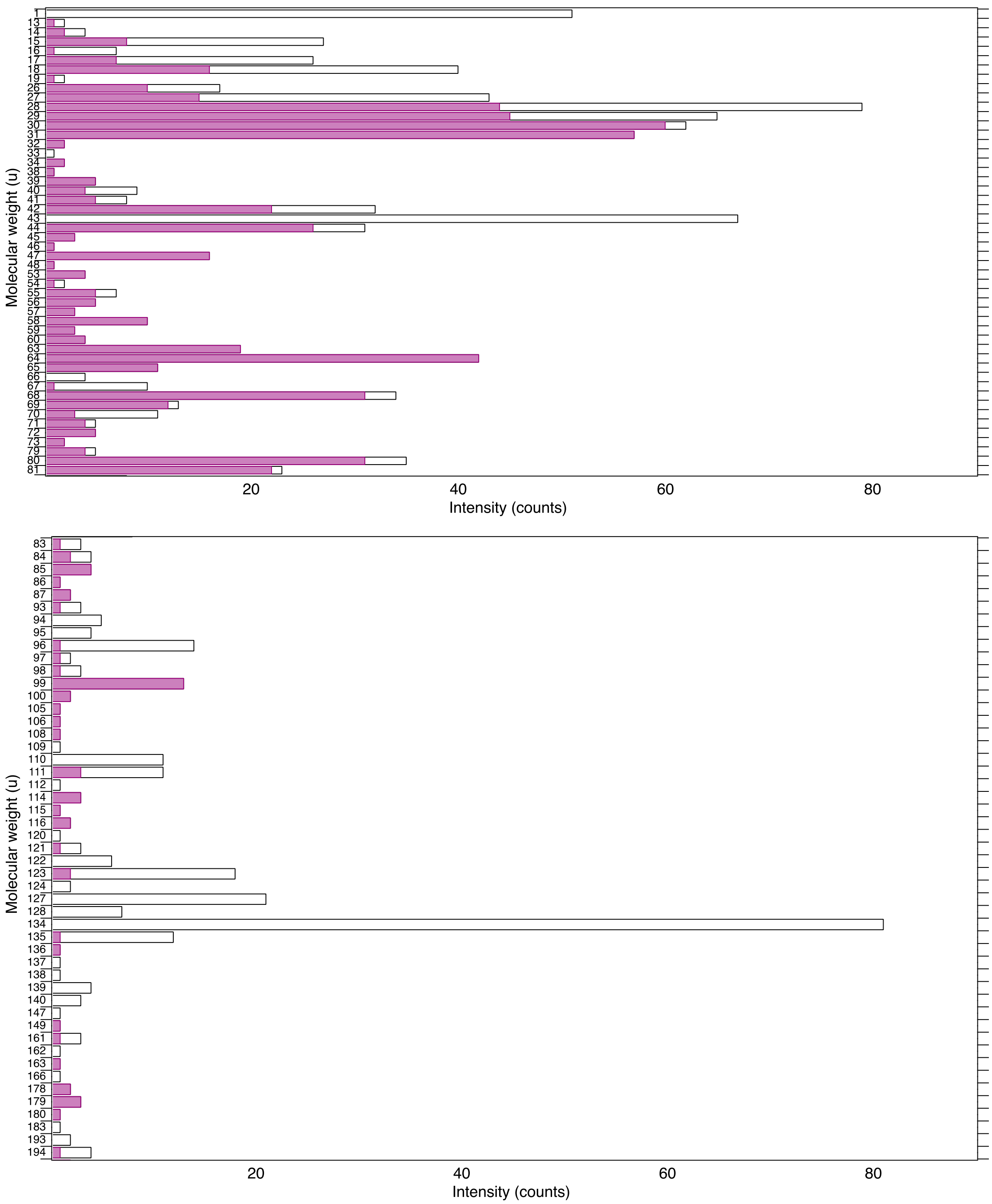

Figure S4: Fragments recorded from simulations at +9 charge at masses between 1 and 194 u. Fragments containing at least one atom from the backbone is shown in purple. For example, no fragments at mass 134 originate from the backbone, while all fragments recorded at 31 comes from backbone breakage.

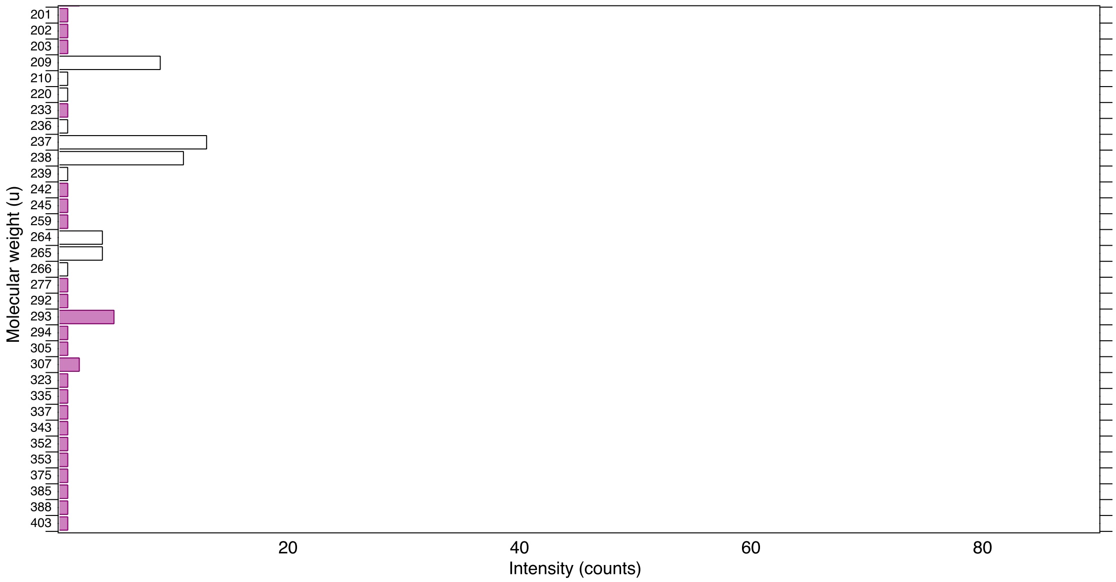

Figure S5: Fragments recorded from simulations at +9 charge at masses between 201 and 403 u. Fragments containing at least one atom from the backbone is shown in purple. For example, no fragments at mass 238 originate from the backbone, while all fragments recorded at 293 comes from backbone breakage.

Fragment	mass (u)	+3 (NoI)	+3 (wI)
H	1.008	24	9
H_3C	15.035	3	1
HO	17.007	4	3
H_3N	17.03	2	-
H_2O	18.015	21	21
H_2C_2	26.038	1	3
HCN	27.026	-	2
H_3C_2	27.046	2	2
CO	28.01	19	26
H_4C_2	28.053	-	2
HCO	29.018	13	14
H_2CO	30.026	61	44
H_3CO	31.034	9	15
H_4CO	32.041	1	-
H_2C_2N	40.044	-	1
H_4C_3	40.064	3	-
CNO	42.017	2	3
H_2C_2O	42.037	4	12
HCNO	43.025	14	9
H_3C_2O	43.045	7	13
H_7C_3	43.088	-	1
CO_2	44.009	-	1
H_4C_2O	44.052	12	20
H_5C_2O	45.06	-	3
H_2CO_2	46.025	1	-
OP	46.973	-	3
H_3CO_2	47.033	-	1
HOP	47.981	-	1
H_5C_4	53.083	1	-
H_3C_3O	55.056	-	2
H_4C_3O	56.063	1	4
HC_2O_2	57.028	1	-
H_5C_3O	57.071	4	4
$H_2C_2O_2$	58.036	7	6
H_6C_3O	58.079	-	1
$H_3C_2O_2$	59.044	5	1
$H_4C_2O_2$	60.051	5	1
O_2P	62.972	5	4
HO_2P	63.98	15	26
H_2O_2P	64.987	2	7
H_3O_2P	65.995	-	1
C_3NO	66.039	-	1
H_4C_4O	68.074	4	10
H_5C_4O	69.082	3	6
H_6C_4O	70.09	5	6
$H_3C_3O_2$	71.055	2	1
H_7C_4O	71.098	6	6
$H_4C_3O_2$	72.062	4	2
$H_5C_3O_2$	73.07	1	10
$H_6C_3O_2$	74.078	-	1
O_3P	78.971	5	6
$H_3C_4N_2$	79.081	1	1
HO_3P	79.979	24	33
$H_4C_4N_2$	80.089	-	2
H_2O_3P	80.986	1	10
H_3C_4NO	81.073	4	-
H_5C_5O	81.093	2	1
H_3O_3P	81.994	2	3
H_6C_5O	82.101	1	-
H_7C_5O	83.109	-	3
$H_4C_4O_2$	84.073	4	-
H_6C_4NO	84.097	-	1
$H_5C_4O_2$	85.081	2	2
$H_6C_4O_2$	86.089	3	5
$H_7C_4O_2$	87.097	-	3
$H_3C_2O_2P$	90.017	-	1
H_2CO_3P	92.997	1	1
$H_5C_5N_2$	93.108	-	1
H_3CO_3P	94.005	1	-
$H_6C_5N_2$	94.115	-	1
$H_5C_4N_3$	95.103	-	1
$H_4C_4N_2O$	96.088	1	1
H_2O_4P	96.985	2	6
$H_5C_5O_2$	97.092	2	-
H_3O_4P	97.993	7	2
$H_6C_5O_2$	98.1	4	9
H_4O_4P	99.001	-	1
$H_5C_4NO_2$	99.088	1	-
$H_7C_5O_2$	99.108	4	16
$H_8C_5O_2$	100.116	5	7
$H_2C_2O_3P$	105.008	-	1
HCO_4P	107.989	-	1
$H_5C_2O_3P$	108.032	1	1
$HC_4N_2O_2$	109.063	-	1
$H_3C_4N_3O$	109.087	2	-
H_3CO_4P	110.004	3	1
$H_2C_4N_2O_2$	110.071	-	3
$H_4C_4N_3O$	110.094	2	1
$H_3C_4N_2O_2$	111.079	-	2
H_4CO_4P	111.012	-	2
$H_5C_5NO_2$	111.099	1	-
$H_5C_4N_3O$	111.102	7	4
H_5CO_4P	112.02	-	1
$H_5C_5O_3$	113.091	1	-
$H_6C_5O_3$	114.099	2	8
$H_7C_5O_3$	115.107	1	4

Table SII: Part 1 of table of generated fragments observed at C K-edge energies (net charge +3) without doping of iodine (NoI) and with iodine at the thymine base (wI) in Born-Oppenheimer based Molecular Dynamics calculations. The numbers represent the total number of fragments.

Fragment	mass (u)	+3 (NoI)	+3 (wI)
$H_8C_5O_3$	116.115	4	13
$H_9C_5O_3$	117.123	-	1
$H_4C_3O_3P$	119.035	-	1
$H_2C_5N_4$	118.097	1	-
$H_3C_5N_3O$	121.098	-	1
$H_3C_2O_4P$	122.015	-	1
$H_4C_5N_3O$	122.105	3	6
$H_4C_2O_4P$	123.023	-	1
$H_3C_5N_2O_2$	123.09	1	-
$H_5C_6NO_2$	123.11	1	-
$H_5C_5N_3O$	123.113	3	7
$H_5C_2O_4P$	124.031	1	1
$H_4C_5N_2O_2$	124.098	1	-
$H_6C_5N_3O$	124.121	-	2
$H_5C_5N_2O_2$	125.106	3	-
$H_9C_6N_2O$	125.149	-	1
O_4P_2	125.944	1	
$H_6C_5N_2O_2$	126.113	4	-
$H_{10}C_6N_2O$	126.157	1	-
I	126.904	-	12
$H_7C_5N_2O_2$	127.121	1	-
HI	127.912	-	6
$H_3C_5N_5$	133.112	1	-
$H_4C_5N_5$	134.12	3	24
$H_4C_3O_4P$	135.034	-	1
$H_5C_5N_5$	135.128	26	49
$H_5C_3O_4P$	136.042	1	-
$H_6C_6N_3O$	136.132	-	2
$H_6C_5N_5$	136.136	12	3
$H_3C_5N_3O_2$	137.097	-	1
$H_5C_6N_2O_2$	137.117	1	-
$H_7C_6N_3O$	137.14	10	1
$H_7C_5N_5$	137.143	1	1
$H_4C_5N_3O_2$	138.104	4	4
$H_8C_6N_3O$	138.148	-	1
$H_4C_2O_5P$	139.022	1	3
$H_5C_5N_3O_2$	139.112	14	20
$H_5C_2O_5P$	140.03	-	1
$H_6C_5N_3O_2$	140.12	4	5
HOI	143.911	-	1
$H_6C_5O_3P$	145.073	-	1
$H_5C_4O_4P$	148.053	-	1
$H_6C_4O_4P$	149.061	2	-
$H_7C_8NO_2$	149.148	1	-
$H_4C_7NO_3$	150.112	-	1
$H_4C_3O_5P$	151.033	1	-
$H_8C_4O_4P$	151.076	1	1
$H_5C_7NO_3$	151.12	-	1
$H_5C_3O_5P$	152.041	-	1
$H_9C_4O_4P$	152.084	1	-
$H_8C_7N_2O_2$	152.151	4	-

Fragment	mass (u)	+3 (NoI)	+3 (wI)
$H_7C_6N_3O_2$	153.139	-	1
$H_9C_7N_2O_2$	153.159	3	-
$H_7C_6N_2O_3$	155.131	1	-
$H_7C_7N_5$	161.165	2	1
$H_7C_5O_4P$	162.08	2	-
$H_8C_5O_4P$	163.087	1	-
$H_5C_6N_5O$	163.138	1	-
$H_6C_7N_3O_2$	164.142	1	1
$H_6C_6N_5O$	164.146	2	-
$H_8C_8N_2O_2$	164.162	1	-
$H_6C_4O_5P$	165.06	1	1
$H_7C_7N_3O_2$	165.15	-	2
$H_8C_7N_3O_2$	166.158	-	7
$H_8C_4O_5P$	167.075	1	-
$H_9C_7N_3O_2$	167.166	5	1
$H_{10}C_7N_3O_2$	168.173	-	1
$H_6C_5O_5P$	177.071	-	1
$H_7C_8N_3O_2$	177.161	1	1
$H_8C_7N_5O$	178.172	1	-
$H_8C_5O_5P$	179.086	2	1
$H_9C_5O_5P$	180.094	-	4
$H_{10}C_5O_5P$	181.102	-	1
$H_7C_7N_3O_3$	181.149	1	-
$H_9C_8N_2O_3$	181.169	1	-
$H_8C_7N_3O_3$	182.157	1	-
$H_{10}C_8N_2O_3$	182.177	1	-
$H_8C_8N_5O$	190.183	-	1
$H_9C_8N_5O$	191.191	2	-
$H_8C_9N_2O_3$	192.172	1	-
$H_{10}C_8N_5O$	192.199	1	-
$H_{10}C_6O_5P$	193.113	1	-
$H_8C_8N_3O_3$	194.168	2	-
$H_{10}C_9N_2O_3$	194.188	2	-
$H_8C_5O_6P$	195.085	-	3
$H_9C_8N_3O_3$	195.176	3	3
$H_9C_5O_6P$	196.093	-	3
$H_{10}C_8N_3O_3$	196.183	4	2
$H_{11}C_8N_3O_3$	197.191	2	-
$H_{12}C_8N_3O_3$	198.199	1	-
$H_4C_5N_3O_4P$	201.076	1	-
$H_8C_9N_5O$	202.194	1	1
$H_9C_9N_5O$	203.202	4	4
$H_8C_9N_4O_2$	204.187	1	-
$H_{10}C_9N_5O$	204.21	1	-
$H_{10}C_{10}N_2O_3$	206.199	1	-
$H_9C_6O_6P$	208.104	-	1
$H_{10}C_9N_3O_3$	208.194	1	1
$H_{12}C_{10}N_2O_3$	208.214	1	-
$H_2C_3N_2OI$	208.966	-	1
$H_{11}C_9N_3O_3$	209.202	1	1
$H_{13}C_{10}N_2O_3$	209.222	1	-

Table SIII: Part 2 of table of generated fragments observed at C K-edge energies (net charge +3) without doping of iodine (NoI) and with iodine at the thymine base (wI) in Born-Oppenheimer based Molecular Dynamics calculations. The numbers represent the total number of fragments.

Fragment	mass (u)	+3 (NoI)	+3 (wI)
$H_{11}C_6O_6P$	210.12	-	1
$H_{10}C_9N_2O_4$	210.187	1	-
$H_{12}C_9N_3O_3$	210.21	1	1
$H_{12}C_6O_6P$	211.128	-	1
$H_{12}C_9N_2O_4$	212.202	1	-
$H_9C_7NO_5P$	218.123	1	-
$H_9C_9N_5O_2$	219.201	1	-
$H_{10}C_9N_5O_2$	220.209	1	2
$H_{10}C_9N_3O_4$	224.193	1	-
$H_{12}C_{10}N_2O_4$	224.213	1	-
$H_{11}C_9N_3O_4$	225.201	3	1
$H_{13}C_{10}N_2O_4$	225.221	1	-
$H_{10}C_9N_2O_5$	226.186	-	1
$H_{10}C_{10}N_5O_2$	232.22	1	1
$H_{11}C_{10}N_5O_2$	233.228	1	6
$H_{12}C_{10}N_5O_2$	234.236	3	3
$H_{10}C_{11}N_2O_4$	234.209	-	1
$H_{13}C_{10}N_5O_2$	235.243	1	-
$HC_4N_2O_2I$	235.968	-	3
$H_2C_4N_2O_2I$	236.976	-	12
$H_3C_4N_2O_2I$	237.983	-	19
$H_4C_4N_2O_2I$	238.991	-	1
$H_{14}C_{10}N_2O_5$	242.228	1	-
$H_{10}C_5O_7P_2$	244.074	-	1
$H_9C_8N_2O_5P$	244.141	1	-
$H_{10}C_8N_2O_5P$	245.149	1	-
$H_{12}C_{10}N_5O_3$	250.235	2	-
$H_{13}C_{10}N_5O_3$	251.242	2	-
$H_{14}C_{10}N_5O_3$	252.25	2	-
$H_2C_4N_2O_3I$	252.975	-	1
$H_8C_8N_3O_5P$	257.14	1	-
$H_7C_8N_2O_6P$	258.124	-	1
$H_3C_6N_2O_2I$	262.005	-	1
$H_9C_7N_3O_6P$	262.135	-	1
$H_5C_6N_2O_2I$	264.021	-	2
$H_6C_6N_2O_2I$	265.029	-	1
$H_{14}C_9O_7P$	265.176	-	1
$H_3C_5N_2O_3I$	265.993	-	1
$H_{16}C_9O_7P$	267.191	-	1

Fragment	mass (u)	+3 (NoI)	+3 (wI)
$H_9C_8N_5O_4P$	270.162	1	-
$H_{11}C_9N_2O_6P$	274.166	1	-
$H_{10}C_5O_9P_2$	276.072	-	3
$H_9C_8N_2O_7P$	276.139	-	1
$H_{10}C_{11}N_5O_4$	276.229	-	1
$H_6C_7N_2O_2I$	277.04	-	1
$H_{10}C_8N_2O_7P$	277.147	-	1
$H_{15}C_{10}O_7P$	278.194	1	-
$H_{15}C_9N_2O_6P$	278.198	1	-
$H_4C_6N_2O_3I$	279.012	-	1
$H_9C_{10}N_2O_6P$	284.162	1	-
$H_{13}C_{10}N_2O_6P$	288.193	2	-
$H_{10}C_9N_2O_7P$	289.158	-	1
$H_{12}C_{10}NO_7P$	289.178	1	-
$H_{13}C_9N_3O_6P$	290.189	2	-
$H_{15}C_{10}N_2O_6P$	290.209	2	-
$H_7C_8NO_3I$	292.051	-	1
$H_6C_7N_2O_3I$	293.039	-	1
$H_{12}C_{10}N_5O_4P$	297.207	2	-
$H_{10}C_9N_5O_5P$	299.18	1	-
$H_{10}C_{10}N_2O_7P$	301.169	-	1
$H_{11}C_{10}N_2O_7P$	302.176	1	-
$H_{12}C_{10}N_2O_7P$	303.184	1	-
$H_{13}C_{10}N_2O_7P$	304.192	8	-
$H_6C_8N_2O_3I$	305.05	-	3
$H_{14}C_{10}N_2O_7P$	305.2	2	-
$H_7C_8N_2O_3I$	306.058	-	1
$H_{13}C_9N_3O_7P$	306.188	1	-
$H_{11}C_{10}N_5O_5P$	312.199	1	-
$H_{12}C_{10}N_5O_5P$	313.206	2	-
$H_{13}C_{10}N_5O_5P$	314.214	2	-
$H_{15}C_{14}N_5O_4$	317.301	1	-
$H_4C_7N_4O_3I$	319.037	-	1
$H_{13}C_7O_{10}P_2$	319.116	-	1
$H_6C_8N_2O_4I$	321.049	-	2
$H_{14}C_{10}N_2O_8P$	321.199	1	-
$H_{13}C_{10}N_5O_6P$	330.213	3	-
$H_{14}C_{10}N_5O_6P$	331.221	3	-
$H_5C_8N_4O_3I$	332.055	-	1

Table SIV: Part 3 of table of generated fragments observed at C K-edge energies (net charge +3) without doping of iodine (NoI) and with iodine at the thymine base (wI) in Born-Oppenheimer based Molecular Dynamics calculations. The numbers represent the total number of fragments.

Fragment	mass (u)	+3 (NoI)	+3 (wI)
$H_{10}C_9N_2O_4I$	337.091	-	2
$H_6C_7NO_5PI$	342.004	-	1
$H_{15}C_{11}N_5O_6P$	344.24	1	-
$H_{15}C_{11}N_3O_8P$	348.224	1	-
$H_{17}C_{12}N_2O_8P$	348.244	1	-
$H_8C_9N_2O_5I$	351.075	-	1
$H_{10}C_9N_2O_5I$	353.09	-	1
$H_{15}C_{10}N_2O_8P_2$	353.181	1	-
$H_{17}C_{10}O_{10}P_2$	359.181	1	-
$H_{15}C_{12}N_2O_9P$	362.228	1	-
$H_{16}C_{12}N_2O_9P$	363.236	1	-
$H_{14}C_{10}N_2O_9P_2$	368.172	3	-
$H_{15}C_{10}N_2O_9P_2$	369.18	1	-
$H_{12}C_{12}N_5O_7P$	369.226	-	1
$H_7C_7N_2O_6PI$	373.018	-	1
$H_{16}C_{12}N_5O_7P$	373.258	1	-
$H_{15}C_{10}N_2O_{10}P_2$	385.179	1	-
$H_7C_{10}N_7O_3I$	400.113	-	1
$H_{15}C_{10}N_2O_{11}P_2$	401.178	1	-
$H_7C_{10}N_7O_3I$	400.113	-	1
$H_8C_8N_2O_7PI$	402.035	-	1
$H_9C_8N_2O_7PI$	403.043	-	1
$H_{20}C_{15}N_5O_7P$	413.322	-	1
$H_9C_9N_2O_7PI$	415.054	-	1
$H_{10}C_9N_2O_7PI$	416.062	-	4
$H_{11}C_9N_2O_7PI$	417.07	-	2
$H_{20}C_{15}N_5O_8P$	429.321	-	1
$H_{12}C_9N_2O_8PI$	434.077	-	1
$H_{20}C_{17}N_4O_8P$	439.336	1	-
$H_{18}C_{14}N_2O_{11}P_2$	452.245	2	-
$H_{19}C_{14}N_2O_{11}P_2$	453.253	2	-
$H_{22}C_{14}N_2O_{11}P_2$	456.276	1	-
$H_{16}C_{13}N_5O_{10}P_2$	464.239	1	-
$H_{12}C_9N_2O_8P_2I$	465.05	-	1
$H_{20}C_{14}N_2O_{12}P_2$	470.26	1	-
$H_{21}C_{14}N_2O_{12}P_2$	471.268	1	-
$H_{15}C_{12}N_2O_8PI$	473.133	-	1
$H_{12}C_9N_2O_9P_2I$	481.049	-	1
$H_{14}C_9N_2O_9P_2I$	483.065	-	1
$H_{23}C_{18}N_4O_{10}P$	486.369	1	-
$H_{10}C_9N_2O_{10}P_2I$	495.033	-	1
$H_{21}C_{18}N_5O_{10}P$	498.36	1	-
$H_{22}C_{18}N_5O_{10}P$	499.368	1	-
$H_{23}C_{18}N_5O_{10}P$	500.376	1	-
$H_{24}C_{15}N_2O_{13}P_2$	502.301	1	-
$H_{26}C_{18}N_5O_{10}P$	503.399	1	-
$H_{23}C_{19}N_7O_8P$	508.402	1	-
$H_{22}C_{15}N_5O_{11}P_2$	510.308	-	1
$H_{24}C_{19}N_5O_{10}P$	513.394	1	-
$H_{13}C_9N_2O_{11}P_2I$	514.055	-	1
$H_{26}C_{19}N_5O_{11}P$	531.409	1	-
$H_{24}C_{20}N_7O_9P$	537.42	1	-
$H_{22}C_{17}N_4O_{13}P_2$	552.321	1	-
$H_{28}C_{18}N_3O_{13}P_2$	556.372	1	-
$H_{16}C_{15}N_5O_9PI$	568.193	-	1
$H_{23}C_{19}N_7O_{11}P_2$	587.373	1	-
$H_{25}C_{19}N_5O_{14}P_2$	609.372	1	-
$H_{22}C_{17}N_5O_{10}PI$	614.261	-	1
$H_{27}C_{20}N_7O_{12}P_2$	619.414	1	-
$H_{21}C_{18}N_5O_{10}PI$	625.264	-	1
$H_{27}C_{20}N_7O_{13}P_2$	635.413	1	-
$H_{20}C_{18}N_7O_9PI$	636.271	-	1
$H_{29}C_{21}N_6O_{13}P_2$	635.433	1	-
$H_{33}C_{24}N_7O_{14}P_2$	705.503	1	-
$H_{38}C_{29}N_{10}O_{16}P_2$	844.615	1	-

Table SV: Part 4 of table of generated fragments observed at C K-edge energies (net charge +3) without doping of iodine (NoI) and with iodine at the thymine base (wI) in Born-Oppenheimer based Molecular Dynamics calculations. The numbers represent the total number of fragments.
